# Predictive analysis of long non-coding RNA expression profiles in diffuse large B-cell lymphoma

**DOI:** 10.18632/oncotarget.15571

**Published:** 2017-02-21

**Authors:** Danxia Zhu, Cheng Fang, Xiaodong Li, Yiting Geng, Ruiqi Li, Chen Wu, Jingting Jiang, Changping Wu

**Affiliations:** ^1^ Department of Oncology, The Third Affiliated Hospital of Soochow University, Changzhou 213003, China; ^2^ Department of Tumor Biological Treatment, The Third Affiliated Hospital of Soochow University, Changzhou 213003, China

**Keywords:** diffuse large B cell lymphoma, long noncoding RNAs, microarray

## Abstract

Long non-coding RNAs (lncRNAs) are implicated in many tumors. To find novel targets for study of diffuse large B-cell lymphoma (DLBCL), our team performed genome-wide analyses of lncRNA expression in 5 DLBCL cell lines using the 4*180K Agilent lncRNA Chip system, and in normal B cells. Five lncRNAs were validated by quantitative reverse transcription polymerase chain reaction. The differentially expressed lncRNAs and mRNAs were identified via false discovery rate and fold-change filtering. Potential targets correlated with DLBCL were recognized via gene ontology and pathway analysis. Establishment of the co-expression network was done using Cytoscape. In total, 1053 lncRNAs and 4391 mRNAs were dysregulated in DLBCL cells, being comparing with normal B cells. The results suggested that the expressions of the 5 lncRNAs were consistent with the chip results. Several terms including the cell cycle, apoptosis, B cell receptor and NF-κB signaling pathways were important in the progression of DLBCL. The chromosome locations of a few lncRNAs and the associated coexpressed genes were demonstrated by cis-regulatory gene analyses. The results of trans-analyses showed that multiple transcription factors regulated lncRNA and gene expression. Those outstanding lncRNAs in each group were implicated in the regulation of the TF-lncRNA-target gene network. Our study identified a set of lncRNAs differentially expressed in DLBCL cells.

## INTRODUCTION

Diffuse large B cell lymphoma (DLBCL) is oen of the most common lymphoid malignancies with a fast growing incidence [1]. The administering of the regimen including rituximab plus cyclophosphamide/doxorubicin/vincristine/prednisone (R-CHOP) has been seemed as the standard therapy for the patients with DLBCL, remarkably improves the prognosis [2, 3]. However, a great amounts of patients still suffer from unsatisfactory prognosis [4]. Thus, it is important to investigate novel biomarkers involved in DLBCL development.

Long non-coding RNAs (lncRNAs), which possess a size larger than 200 nucleotides, have been found of pivotal roles in multiple processes. LncRNAs regulate gene expression at post-transcriptional level [5] and modulate transcriptional gene silencing via the chromatin regulation [6, 7]. Dysregulation of lncRNAs is regarding with diseases including cancer [8]. For instance, MALAT1 is attributed to lung cancer [9], HOTAIR to breast cancer [10], and HULC to pancreatic cancer [11]. However, the patterns and biological function of lncRNA expression in DLBCL is still unclear.

Here, our team performed lncRNA chip array assays on DLBCL cells. The function of lncRNA was annotated according to coexpressed genes and the biological process of gene ontology (GO). The associations amongst the lncRNAs were studied by cis- and trans-regulatory gene analysis method. These studies confirmed that aberrant lncRNA expression plays a crucial role in lymphomagenesis.

## RESULTS

### Expression profiles of lncRNA and mRNA in DLBCL

**Figure 1:**
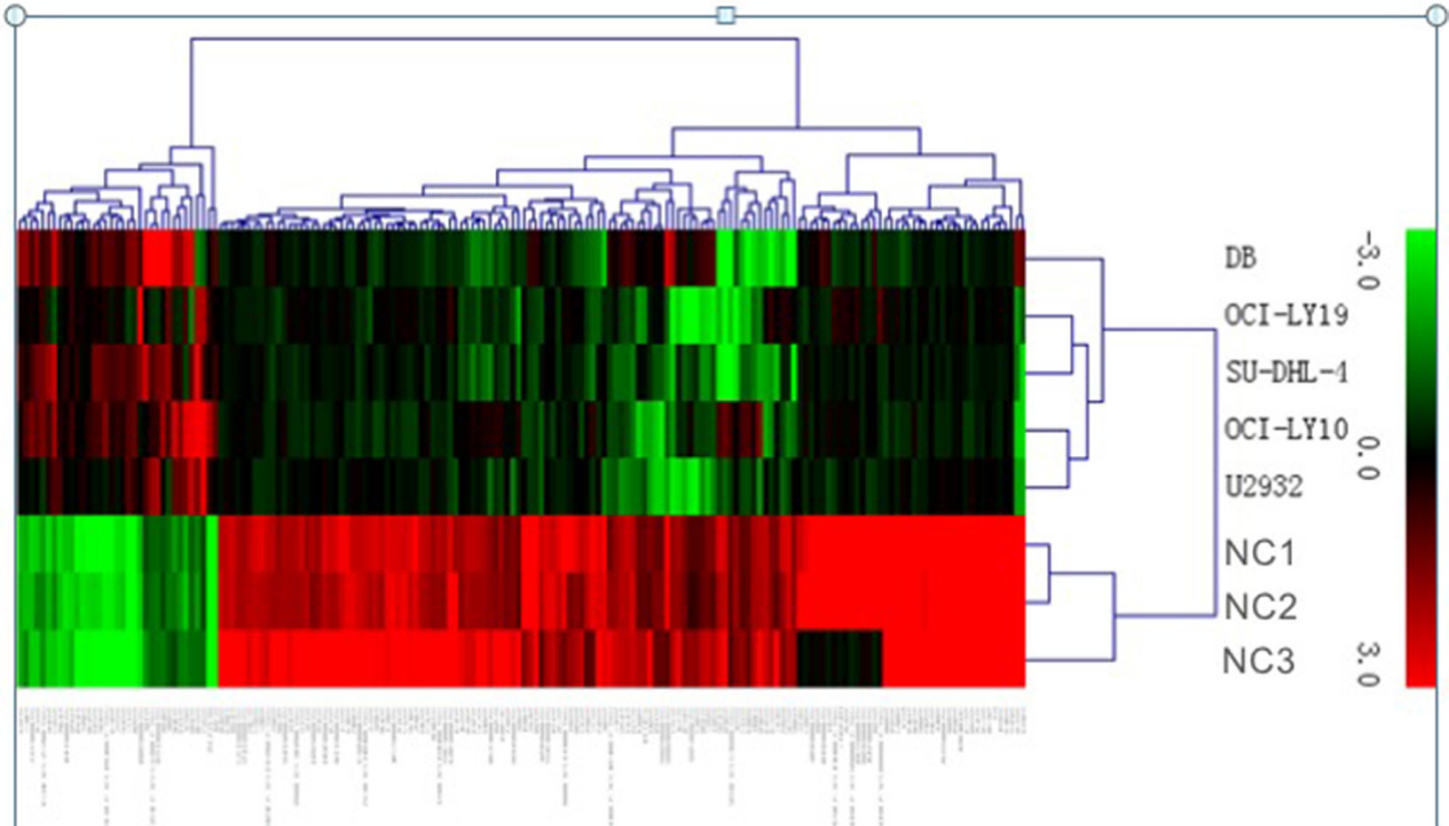
Heat map showing differentially expressed lncRNAs from DLBCL cells compared with three normal B cells Each row represents one cell sample, and each column represents one lncRNA. The relative lncRNA expression is depicted according to the color scale. Red indicates up-regulation; green indicates down- regulation. 3.0, 0, and 3.0 are fold changes in the corresponding spectrum.

LncRNA profiling screened out 1,053 lncRNAs of significant levels of differential expression in DLBCL cell lines comparing with normal B cells (> 1.5-fold change; FDR < 0.05); 416 were significantly upregulated and 637 were significantly downregulated in DLBCL cells. The normalized data of microarray expression were collected to produce heatmaps (Figure 1). Unsupervised hierarchical clustering analyses revealed that systematic variation of 176 lncRNAs expression (> 5.0-fold change; FDR < 0.05) between normal B cells and DLBCL cells. Amongst the dysregulated transcripts of lncRNA, NAALADL2-AS2 was the most upregulated, with an FC of 61.59, whereas NONHSAT120161 was the one with the most downregulated extent, with FC being −52.78. The top 20 differentially expressed lncRNAs identified using microarray analyses are listed as Table 1. Using the same criteria of lncRNAs (> 1.5-fold change; FDR < 0.05), we found that 4,391 mRNA transcripts were dysregulated, with 1,896 upregulated, and 2,495 downregulated (Supplementary Figure 1). The most upregulated and downregulated mRNA transcripts were FAM72D and LYZ, respectively, with FCs of 29.12 and −159.90, respectively.

**Table 1:** Top 20 up-regulated and down-regulated lncRNAs and mRNAs in DLBCL cells compared with normal B cells

Up-regulated lncRNAs		Down-regulated lncRNAs		Up-regulated mRNAs		Down-regulated mRNAs	
lncRNA	FC	lncRNA	FC	mRNA	FC	mRNA	FC
NAALADL2-AS2	61.59	NONHSAT120161	−52.78	FAM72D	29.12	LYZ	−159.90
NONHSAT005757	33.63	NONHSAT077537	−48.07	BCAT1	20.27	ANXA1	−51.29
NONHSAT009310	13.03	XIST	−43.94	FAM72C	19.85	GZMA	−48.81
NONHSAT053548	12.17	NONHSAT097386	−30.09	TOP2A	14.13	CD96	−46.98
NONHSAT132953	10.45	NONHSAT005144	−28.03	ANLN	13.93	CD3G	−44.55
NONHSAT101891	9.29	NONHSAT105043	−26.47	RGS13	13.85	SELL	−37.94
NONHSAT075369	9.05	NONHSAT124144	−23.64	SLC7A11	13.73	PARP15	−36.71
NONHSAT145556	8.84	NONHSAT060917	−23.49	CDK1	13.67	BIN2	−35.92
NONHSAT001763	8.59	NONHSAT011406	−21.51	IGF2BP3	13.60	GBP5	−35.09
NONHSAT097625	8.41	NONHSAT029287	−19.99	FAM72B	13.03	FYB	−34.19

### Microarray data validation by qPCR

**Figure 2:**
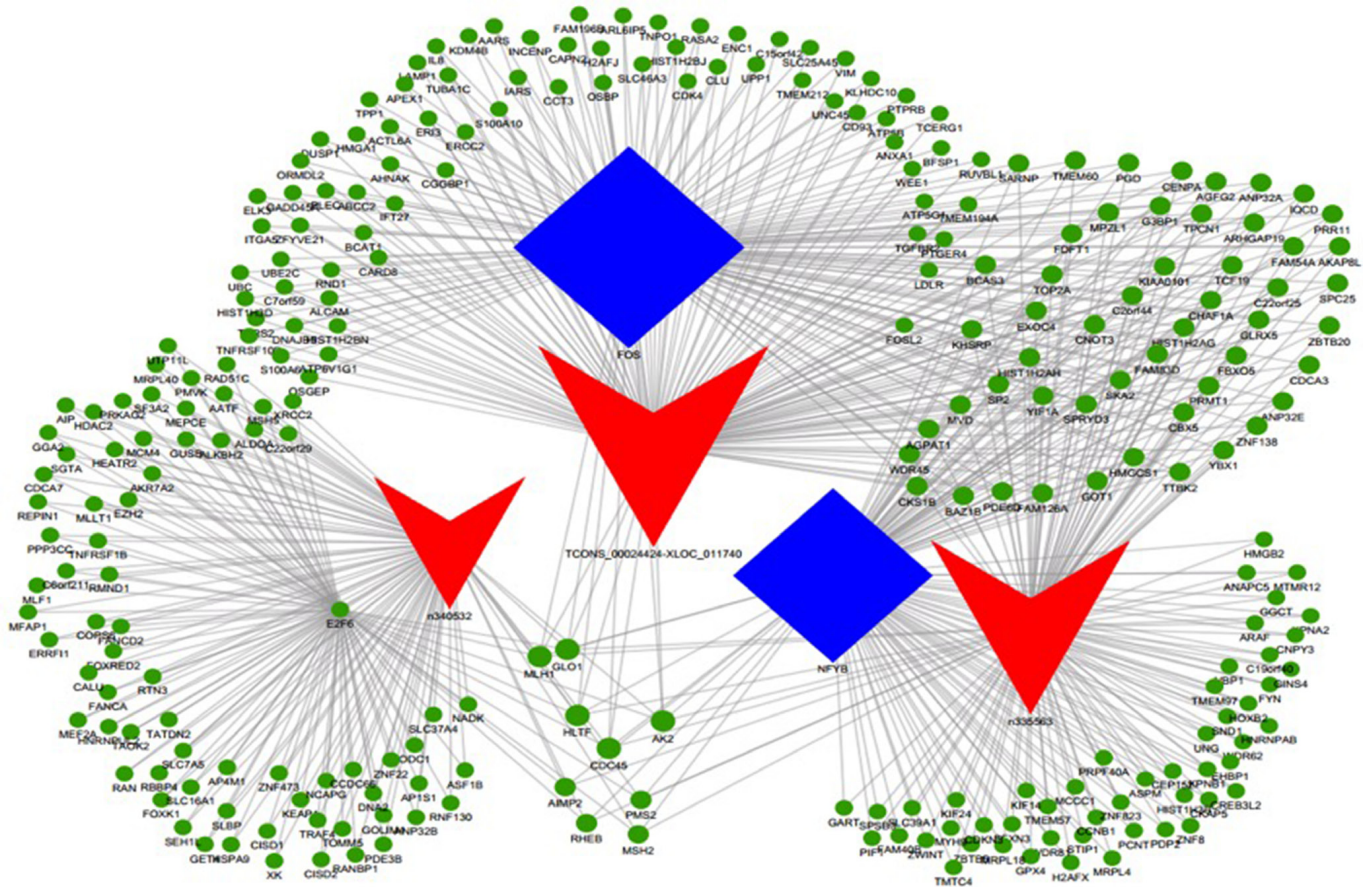
LncRNA-TF-mRNA core network consisting of the top 500 pairs of lncRNA, TF, and mRNA with the most relevance The red triangle represent lncRNAs, blue rectangular represents TFs, and green circle represent mRNAs.

**Figure 3:**
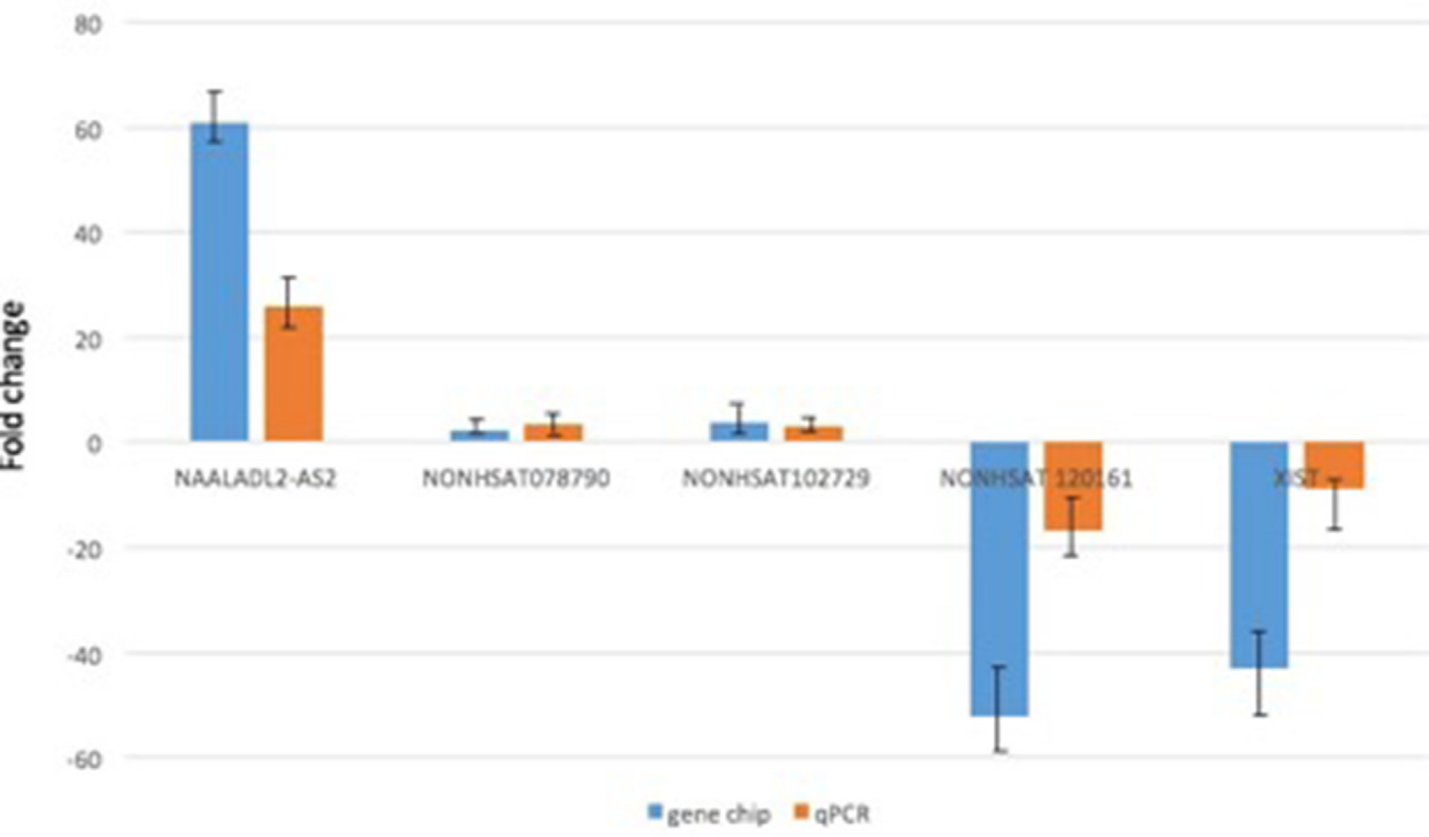
qRT-PCR validation of 5 differentially expressed lncRNAs in 20 DLBCL tumor samples The result showed that expressions of lncRNAs were consistent with the microarray data in lymphoma tissues relative to the normal B cell samples. The heights of the columns in the chart represent the mean fold changes in expression for each of these lncRNAs. Bars represent SEM. Fold change is positive when the expression is upregulated (DLBCL cell/normal B cell) and negative when down-regulated.

On the basis of lncRNA function predictions, 5 lncRNAs (NAALADL2-AS2, NONHSAT 120161, NONHSAT078790, NONHSAT102729, and XIST) were sorted out for further validation of the microarray consistency using qPCR. By analyzing microarray data, we found NAALADL2-AS2 was upregulated mostly and NONHSAT 120161 was downregulated mostly. NONHSAT102729 and NONHSAT078790 were the core lncRNAs in TF-lncRNA network (Figure 2, n335563 and n340532 respectively), and XIST has been extensively studied in other cancers and it is also downregulated in DLBCL cell lines to certain extent [12]. We believe these lncRNAs may involve in the development of DLBCL; therefore, we chosen them to validate. The results demonstrated that lncRNA NAALADL2-AS2, NONHSAT078790, and NONHSAT102729 were upregulated and that XIST and NONHSAT120161 were downregulated in the samples of DLBCL comparing with normal B cells (Figure 3). These qPCR results were consistent with microarray data.

### Coexpression profiles of lncRNA and mRNA and the prediction of lncRNA function

Hundreds of lncRNAs are correlated with thousands of mRNAs. For instance, NAALADL2-AS2 was correlated with 3,998 mRNA transcripts and XIST with 3,820 ones (Supplementary Figure 2). Functions of differentially expressed lncRNA were predicted using GO pathway annotations of their correlated mRNAs. These lncRNAs could be categorized into hundreds of KEGG and GO pathway annotations. Some of the pathways are involved in the pathogenesis of lymphomagenesis, such as cell cycle, cell apoptosis, B cell receptor signaling, and NF-kappa B signaling pathway. Of these, 230 lncRNAs were clustered into regulation of NF-kappa B signaling pathway and 125 were into the B cell receptor signaling pathway. One lncRNA was involved in more than one GO/KEGG pathway regarding with lymphomagenesis. For example, NAALADL2-AS2 is predicted to have regulatory functions in p53, NF-kappa B, JAK-STAT signaling pathways, and in hematopoietic cell lineage.

**Figure 4:**
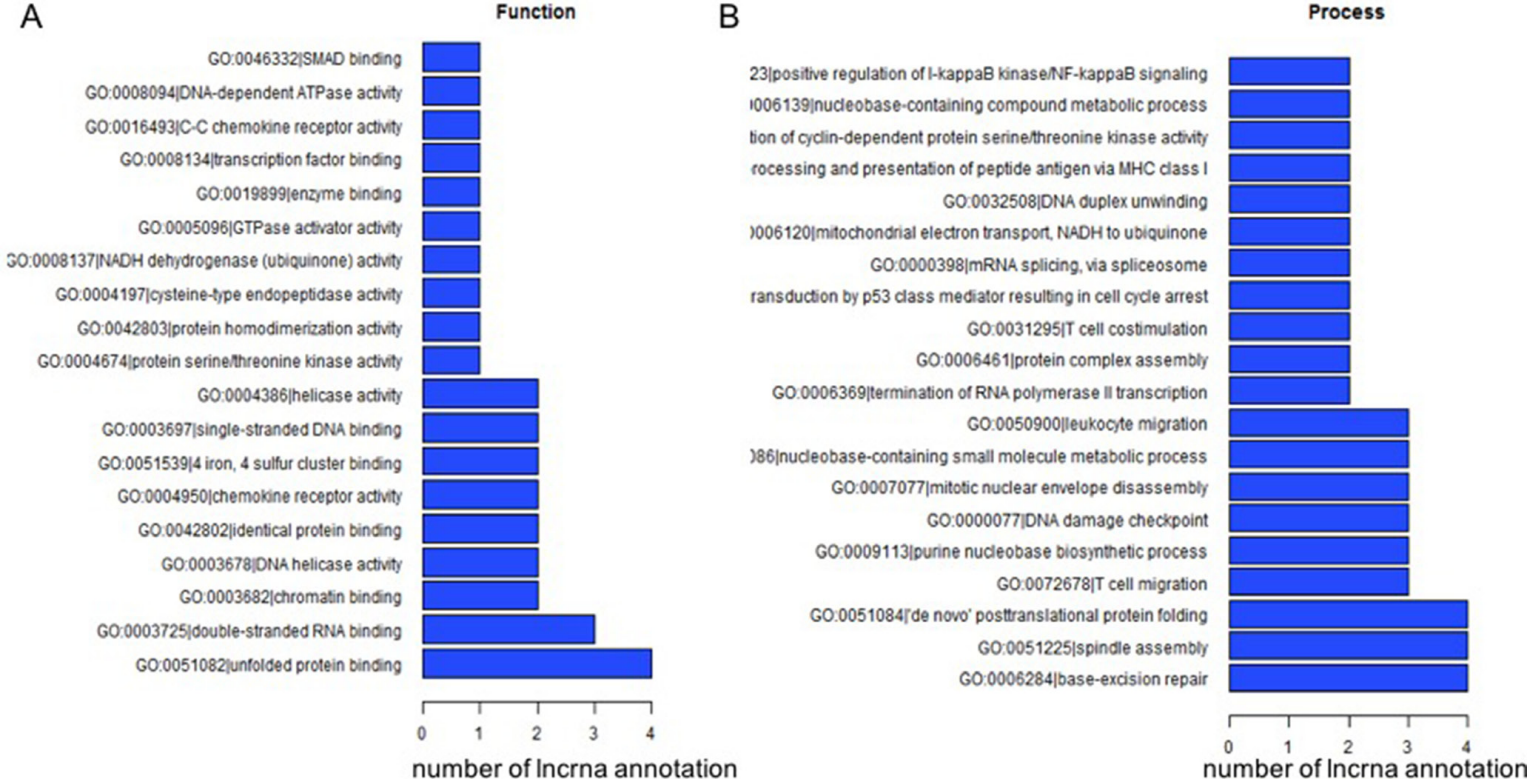
The top 200 GO terms or pathways for the difference lncRNA co-expression genes between the DLBCL cells and normal B cells

Of the complex network for lncRNAs and their corresponding GO annotations of correlated protein coding genes, we summarized the top 200 annotations with the most significance (i.e. the lowest *P* values). The most frequently predicted functions of differentially expressed lncRNAs were unfolded protein binding, double-stranded RNA binding, and chromatin and nucleosome assembly (Figure 4).

### Cis regulation of lncRNA

A few lncRNAs regulate their own transcription in the cis-regulatory genes, and also that of the nearby genes, via recruition of remodeling factors to local chromatin [13]. Thus, we identified the chromosomal coexpression genes 300 kbp upstream and downstream of the differentially expressed lncRNAs to determine potential lncRNA cis-regulatory genes. These genes were considered as potential cis-regulatory genes of the lncRNAs. A data reduction strategy was used for overlaying the genes located at up- or down-stream of lncRNAs and coexpression genes. When comparing DLBCL cells versus normal B cells, 241 lncRNAs had 576 cis-regulatory genes that were identified. Among these, the lncRNAs NONHSAT035752 had 13 cis-regulatory genes, and the lncRNA with the most downregulated expression, lncRNA NONHSAT120161, had four cis-regulatory genes (TRGV10, TRGV1, TRGV3, and TRGV5) (Supplementary Figure 3). We showed that in the top 200 downregulated lncRNAs, 11 were possibly cis-regulated immunoglobulin genes (IGHV, IGLV, IGKV, IGLJ, IGHD, IGHJ) (Supplementary Figure 4). However, the top 200 upregulated expressed lncRNA genes had similar functions.

### Trans-regulation of lncRNA and the construction of the TF-lncRNA-target gene network

Using the threshold of FDR < 0.01, 4,971 lncRNA-TF pairs were found, corresponding to 71 TFs. Most of these potential trans-regulatory lncRNAs were found to participate in pathways regulated by several TFs: NF-YA, NF-YB, STAT1, STAT2, MYC, and E2F6. Among the core network of lncRNA-TF pairs, E2F6 participated in 32 of the 100 pairs, FOS in 26, and NF-YB in 20 pairs.

**Figure 5:**
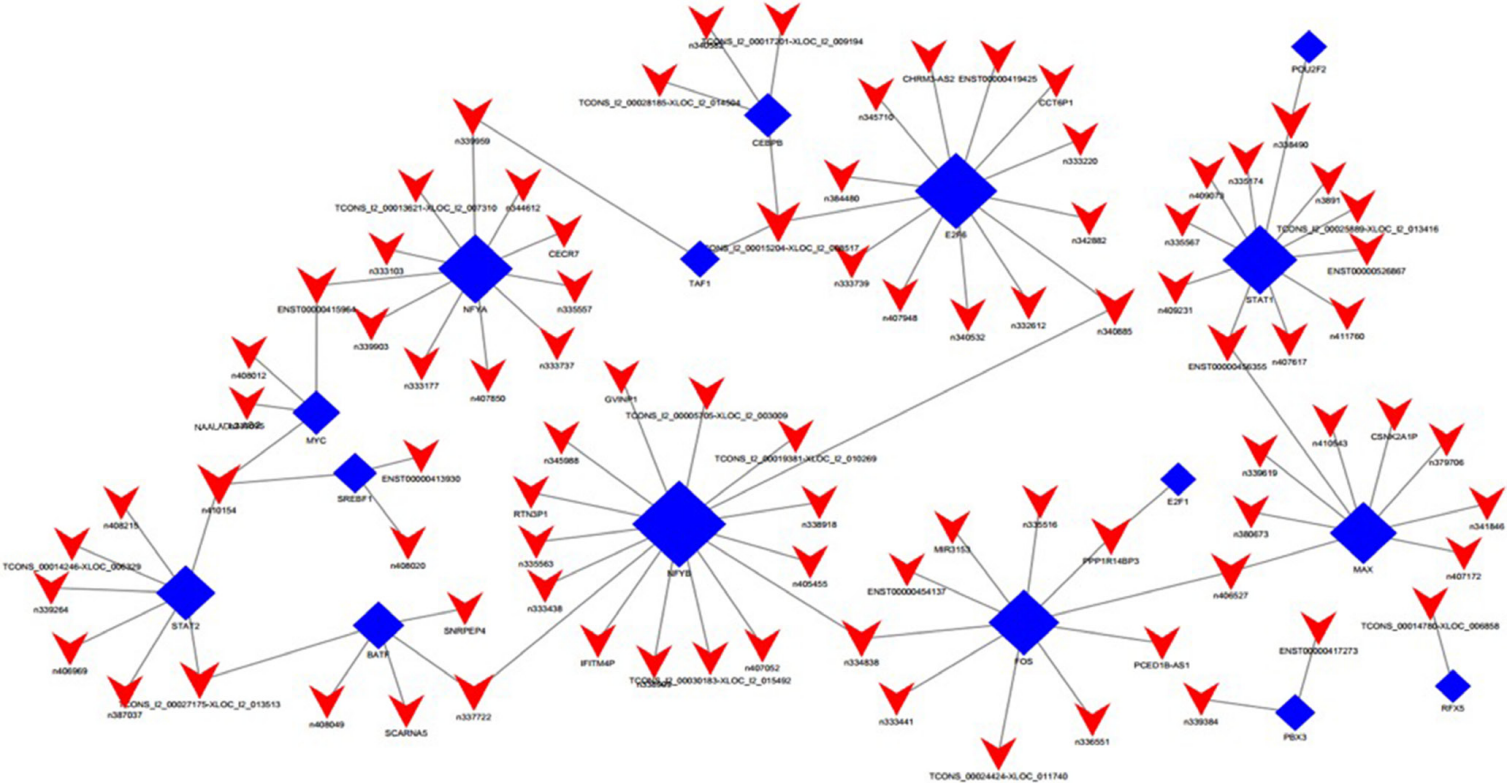
LncRNA-TF core network consisting of the top 100 pairs of lncRNA and TF with the most credentiality Most of the lncRNAs are predicted to be regulated by NF-YA, NF-YB, E2F6, STAT1 and FOS. The solid rectangulars represent TFs, and solid triangle represent lncRNAs; the edges between them mean that the lncRNAs are potentially regulated by the TFs.

The TF-lncRNA two-element network was generated by using Cytoscape software. The TF-lncRNA network was complex due to that numerous aberrant lncRNAs were involved. So we selected the top 100 closest relationships with the TF-lncRNA network to generate a core network map (Figure 5).

Besides, we added the target genes into the TF-lncRNA network to determine the 3-element network correlation. The networks were large and complex; therefore, we selected the top 500 relationships amongst lncRNAs, TFs, and target genes to generate the core network map (Figure 2). Figure 2 shows the core TF-lncRNA-target gene relationship of DLBCL cells vs. controls, with 3 lncRNAs with disrupted expression (TCONS_00024424-XLOC_011740, NONHSAT102729, and NONHSAT078790), 326 target genes and three core TFs (FOS, NF-YB, and E2F6) in the core map (Figure 2). For MYC-NAALADL2-AS2 in Figure 5, predicted target genes for NAALADL2-AS2, such as MTHFD2, were coexpression genes for NAALADL2-AS2 (Supplementary Figure 2). MYC might regulate the expression of lncRNA NAALADL2-AS2 and the target genes like MTHFD2.

## DISCUSSION

There have not been many studies of the expression profiles of lncRNAs in DLBCL or studies predicating the correlation between lncRNA expression and clinicopathological features and prognosis in DLBCL [14, 15, 16].

In the current research, for the first time, we detected genome-wide lncRNA expression patterns within DLBCL cell lines comparing with normal B cells using microarray technologies, and also investigated their potential functions based on analyzing their correlated protein coding mRNAs. The results revealed that 1,053 lncRNA and 4,391 mRNA transcripts were dysregulated. In addition, qRT-PCR was employed to validate the microarray analyses findings, and the results were consistent with the microarray data. These results suggested that there were unique lncRNA expressions in DLBCL cells.

There are several hundreds of lncRNAs correlated with thousands of mRNAs, some of which play crucial roles in progression of DLBCL by influencing the mRNAs in cis- and/or trans-regulatory manner(s). This is to be expected because of the pervasive transcription of lncRNAs in the human transcriptome, their diverse but central roles as key players in cell biology, and the nature of DLBCL as a multifactorial disease involving complicated biological pathways and networks [15, 16].

However, merely a small proportion of known lncRNAs have functional annotations. The most usual method to predict the functions is through interrogating their correlated protein coding genes and related biological pathways, a method called “guilt by association” [17]. In our study, we found that thousands of mRNA transcripts were correlated with the differentially expressed lncRNAs. By clustering the mRNAs into the GO/KEGG pathway annotations, we predicted possible functions of the lncRNAs. As shown in Figure 4, the top predicted pathways involving the lncRNAs are unfolded protein binding, double-stranded RNA binding, and chromatin and nucleosome assembly, which correspond well with the central roles lncRNAs play in the epigenetic regulation of the genome [18]. Among the hundreds of pathways involving lncRNA clusters with their correlated protein coding genes, some were key pathways in the pathogenesis and development of lymphomagenesis. Multiple therapies targeting the NF-κB pathway or the B-cell receptor signaling pathway are under evaluation and are the most promising for ABC-DLBCL, where these pathways are constitutively active. Thus, these groups of lncRNAs may potentially play an important role in lymphomagenesis, and may provide potential targets for future treatment of DLBCL.

By definition, noncoding RNAs have the intrinsic property of cis-regulation, comparing with mRNAs, which can only function through dissociation, transportation, and translation [17]. It has also been suggested that lncRNAs function through cis-regulation of nearby protein coding genes as a common mechanism [19]. In the current research, 241 lncRNA transcripts were predicted to participate in cis-regulation of their nearby protein coding genes. Interestingly, though most of them were uncharacterized, there were a series of lncRNAs (ENST00000425181, ENST00000385099, ENST00000385100,XLOC_000986, ENST00000385097, XLOC_l2_005190, and NONHSAT072333) that possibly cis-regulate immunoglobulin genes (IGHV, IGHD, IGHJ, IGKV, and IGLV). The immunoglobulin genes play important roles in B cell development and many B-cell lymphomas contain Ig gene rearrangement and always express a unique clonal surface Ig which provides a specific tumor marker, suggesting that these lncRNAs may regulate Ig gene rearrangements.

Though a few lncRNAs are confirmed as cis regulators, the majority of functionally characterized ones are trans-regulators [20]. Comparing to cis regulation, trans-regulating lncRNAs dissociate from the synthesis site, impact on distant genes, as well as act in regulatory networks. We found that in the core network of lncRNA-TF pairs, the lncRNAs could be categorized into pathways regulated by NF-YA, NF-YB, STAT1, STAT2, MYC, or E2F6.

NF-Y, a heterotrimeric transcription factor, consists of e subtypes, NF-YA, -YB, and -YC, all of which are required for DNA binding at CCAAT-containing promoters [21]. Numerous *in vitro* studies have implicated NF-Y involved in the regulation of the cell cycle. E2F6 is a member of the E2F TF family, and is proposed to be primarily a transcription repressor. Emerging studies have identified hundreds of E2F6 targets involved in a wide range of functions [22]. MYC possesses a lot of physiological functions. MYC oncogene rearrangements can be identified in ~10% of patients with DLBCL. A MYC rearrangement correlates with a worse outcome in DLBCL patients treated with R-CHOP [23]. More common than MYC translocation, overexpression of MYC protein due to upregulation by additional mechanisms can be measured by immunohistochemical techniques in 25%–30% of DLBCL patients. The lncRNAs that are predicted to be involved in the pathways regulated by MYC are thus candidate participants in the pathogenesis of DLBCL. Based on the lncRNA-TF-mRNA analyses (Figure 2), our data showed that MYC was recruited by > 200 lncRNAs in pathway analyses, and NAALADL2-AS2 was the most upregulated lncRNA that could be regulated by MYC, suggesting that there were cooperating patterns of lncRNAs involved in the development of DLBCL.

Our study has some limitations. Our work predicted only lncRNA functions indirectly within network and pathway analyses of relevant protein coding genes. This is due to most of the discovered lncRNAs have not yet been functionally annotated [24], therefore, the “guilt by association” approach for hypotheses generation is an important primary step for further functional studies, such as loss/gain of function experiments [25].

In conclusion, our results demonstrated that 1,053 lncRNAs were differentially expressed i DLBCL cells and normal B cells. Multiple lncRNAs may be involved in biological pathways related with DLBCL via cis- and/or trans-regulating target protein coding genes. Our research therefore provides basis for further functional studies focusing on lncRNAs regarding with DLBCL as diagnostic and therapeutic targets.

## MATERIALS AND METHODS

### Cell subpopulations and cell lines

Normal B cells (CD19^+^) were purified from tonsillar materials (taken from 9 individuals) by positive immunomagnetic selection (Miltenyi Biotec, Bisley, UK). The Ethics Committee of the The Third Affiliated Hospital of Soochow University had approved the protocol of this study (ML25435). Two ABC-like DLBCL cell lines (OCI-ly10 and U-2932) and three GCB-like DLBCL cell lines (OCI-ly19, SU-DHL-4, and DB) were cultured in RPMI 1640 media containing 10% fetal calf serum (Invitrogen, Paisley, UK).

### The ncRNA and mRNA microarray expression profiling

Total RNA was extracted with TRIzol reagent (Invitrogen, Carlsbad, CA, USA) according to the manufacturer's protocol. Total RNAs was quantified by the NanoDrop^™^ ND-2000 (Thermo Fisher Scientific, Scotts Valley, CA, USA) and the RNA integrity was assessed using the Agilent Bioanalyzer 2100 (Agilent Technologies, Santa Clara, CA, USA). The microarray was analyzed using the OE Biotech Human lncRNA chip software, version 2.0 (4*180K; OE Biotech, Shanghai, China), containing 46,506 lncRNAs and 30,656 mRNAs collected from eight authoritative databases, including Agilent_ncRNA, lncRNAdb, GencodeV13, H-invDB, NONCODEv3.0, RefSeqUCR, and UCSC_lncRNAs transcripts. After hybridization and washing, processed slides were scanned with the Agilent G2505C microarray scanner (Agilent Technologies). Raw data were extracted using Feature Extraction (version 10.7.1.1; Agilent Technologies). Next, quantile normalization and subsequent data processing were done using Genespring software (version 12.0; Agilent Technologies). The microarray profiling was conducted in the laboratory of the OE Biotech Company (Shanghai, China).

### Microarray results for the analysis and prediction of the lncRNA functions

#### Differential expression levels of mRNAs and lncRNAs from microarrays

After quantile normalization, raw signals from microarrays were log_2_ transformed. Differential expression of an mRNA or lncRNA was defined by an absolute value of fold change (FC) > 1.5. The false discovery rate (FDR) was used to evaluate the significance of the P-value and an FDR<0.05 was considered to be significant. The differentially expressed mRNAs were submitted to the DAVID database (http://david.abcc.ncifcrf.gov) to be classified into different GO and Kyoto Encyclopedia of Genes and Genomes (KEGG) annotation groups.

### Coexpression of lncRNAs with mRNAs and functional annotation

Most of the lncRNAs in the current databases have not yet been functionally annotated. Thus, the prediction of their functions is based on the functional annotations of their coexpressed mRNAs. This method was originally described by Guttman et al. [28]. In brief, for every dysregulated lncRNA, the Pearson correlation coefficient (PCC) of its expression comparing with that of each dysregulated mRNA was calculated to find its coexpressed mRNAs, with PCC > 0.8 or < 0.8 with a *P* value of PCC < 0.01 being statistically significant. The functional enrichment analyses of the coexpressed mRNAs were conducted using the hypergeometric cumulative distribution function, and the GO/KEGG pathway annotations were assigned to the lncRNA as its predicted functions. The threshold of statistical significance was set at false discovery rate (FDR) < 0.01.

### Cis-regulatory gene analyses of lncRNAs and coexpressed genes

The gene locations for different lncRNAs on the chromosome were determined. Subsequently, the common lncRNA coexpressed genes were intersected to identify the genes 300 kbp upstream or downstream of the lncRNAs as potential cis-regulatory genes. The schematic shows the chromosome location of the lncRNAs and cis-regulatory genes (Supplementary Figure 3).

### The TF-lncRNA network

The lncRNA sequences were mapped to the genome with the Sanger database. Jemboss software was used to examine the alignment of the lncRNA and the putative transcription factor binding sequences. The genome browser database was used to build the network describing the relationships between transcription factors and lncRNAs. An adjacency matrix was implemented in Java according to the binding of lncRNAs and transcription factors. The core transcription factor is the most important center in the network, with the highest degree of expression [29, 30]. Pearson correlation analyses were used to measure the regulatory ability of transcription factors by calculating the correlation between transcription factors and lncRNAs. The TF and lncRNA relationship was generated using Cytoscape software (http://www.cytoscape.org). The solid rectangles and solid triangles indicate the enriched lncRNAs or TFs, and the larger size indicates increasing enrichment.

### TF-lncRNA-gene network

The TF-lncRNA-gene network was constructed based on the interactions of lncRNAs and target coexpression genes as previously described [31]. To categorize lncRNAs that potentially have trans-regulation functions, the lncRNA target predictions were superimposed onto the lncRNA-TF-mRNA correlation network using Cytoscape software. Therefore, we selected the top 500 closest associations with the “TF-lncRNA-gene” network to produce the core network map. In this network, red triangles represent lncRNAs, blue rectangles represent TFs, and green circles represent mRNAs.

### Real-time quantitative reverse transcription-polymerase chain reaction (qRT-qPCR)

Twenty tumors from DLBCL patients (13 ABC-DLBCL, 7 GCB-DLBCL) and 10 reactive lymphatic nodes (RLN) were used to verify lncRNA expression by qRT-qPCR. β-actin was used as an internal control. Using the Trizol reagent, total RNA was extracted from fresh frozen samples. The primers are listed in Table 2. The qPCR was performed using the SYBR Green (Takara Bio Inc., Dalian, China) dye detection method on the ABI 7300 PCR instrument under default conditions: 95°C for 10 sec, 40 cycles of 95°C for 5 s, and 55°C for 30 s. The relative gene expression levels were analyzed by the 2-ΔCt method, where ΔCt = Cttarget − Ctβ-actin.

**Table 2:** Primers used for qRT-PCR analysis of lncRNA levels

Target ID	Primers	Product Length (bp)	Tm (°C)
β-actin	Forward: AGCGAGCATCCCCCAAAGTT Reverse: GGGCACGAAGGCTCATCATT	265	60
NAALADL2-AS2	Forward: GAGCCAACTGGGATAAAGAA Reverse: AGGAAGGCAACTGTCACTCT	145	60
NONHSAT120161	Forward: ACCTGAAACGTCTACATCCA Reverse: GTTGTTCCACTGCCAAAGAG	116	60
NONHSAT078790	Forward: CGGATGAAAGTACGGGTCG Reverse: GAGCTGCCTGCAAATGGTC	167	60
NONHSAT102729	Forward: AGAAGCCACAGGGAAAATCA Reverse: CTCTAGCAACACGTCGCAAA	160	60
XIST	Forward: GGATGTCAAAAGATCGGCCC Reverse: GTCCTCAGGTCTCACATGCT	137	60

lncRNAs = long noncoding RNAs, qRT-PCR = quantitative real-time polymerase chain reaction. The table lists the 5 primers used for qRT-PCR.

## SUPPLEMENTARY MATERIALS FIGURES AND TABLES


