# Supplementary material for: Predictive analysis of long non-coding RNA expression profiles in diffuse large B-cell lymphoma
Source: Oncotarget. 2017 Feb 21;8(14):23228–36. doi: 10.18632/oncotarget.15571 (PMC5410299; doi:10.18632/oncotarget.15571)
Supplement: Supplementary file 1 [file oncotarget-08-23228-s001.pdf]

Predictive analysis of long non-coding RNA expression profiles in diffuse large B-cell lymphoma

Supplementary Materials

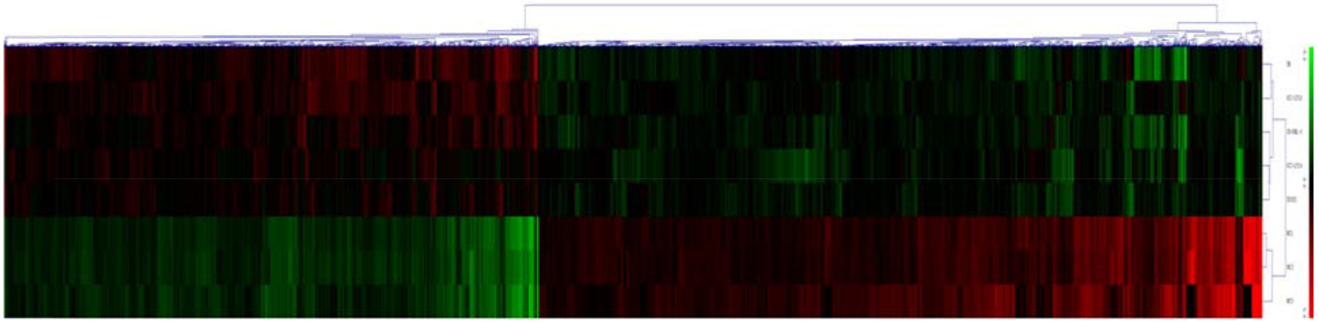

Supplementary Figure 1: Hierarchical clustering of mRNA in DLBCL cell lines and three pooled controls. The level of mRNA expression is color-coded. Red, higher mRNA expression; green, lower mRNA expression.

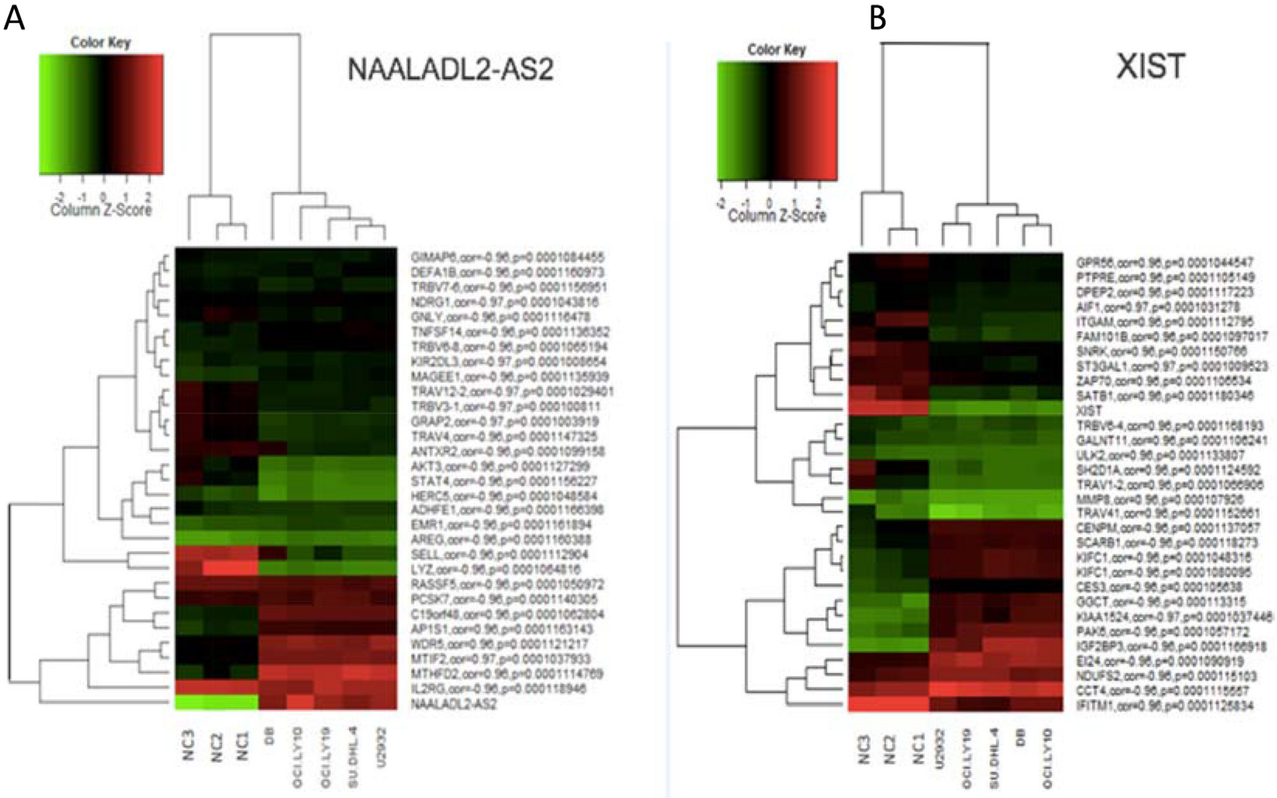

Supplementary Figure 2: For function prediction of lncRNAs, the enriched functional terms were used as the predicted functional term of given lncRNA. The correlated mRNAs of lncRNAs were identified by calculating Pearson Correlation with correlation *P*-value < 0.05. (**A**) The correlated mRNAs of lncRNA NAALADL2-AS2; (**B**) The correlated mRNAs of lncRNA XIST).

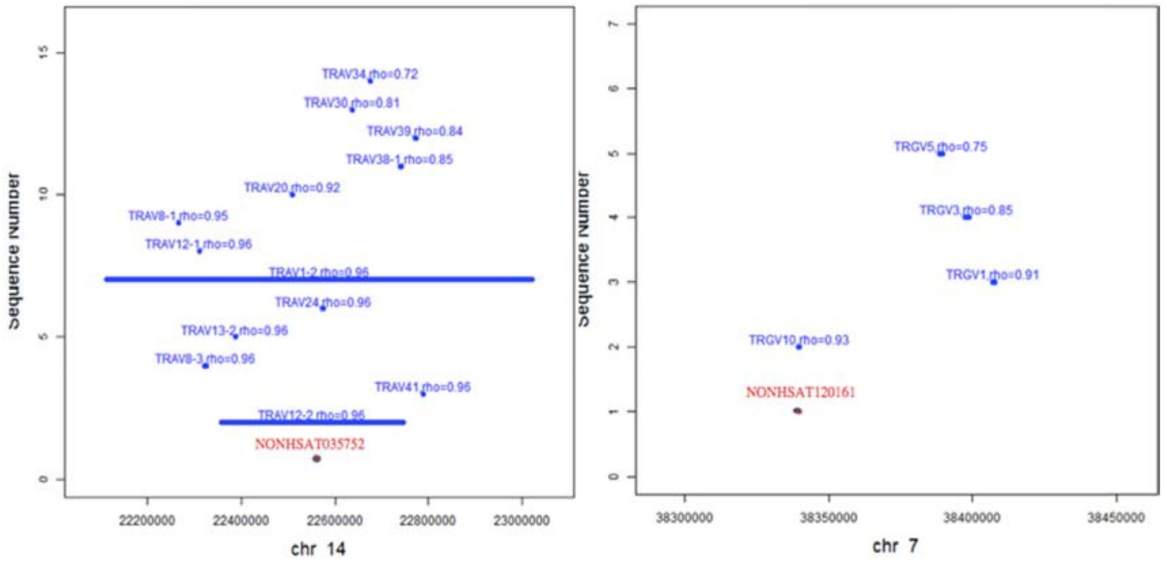

Supplementary Figure 3: Representative lncRNAs and the positions of their 'cis' genes in the chromosome. The X abscissa represents the chromosome genome position, and the Y coordinate represents the correlation coefficient of the lncRNAs and the 'cis' genes, with a greater correlation coefficient corresponding to a higher position. The red line (or points) indicates the lncRNA genome width, and numbers in parentheses indicate the length. The blue line (or points) indicates the location of the encoding genes; rho values for the encoding genes and lncRNA expression correlation coefficients between the values, as well as the P-values for the correlation coefficients are shown. (The results for the lncRNAs NONHSAT035752 and NONHSAT120161 are shown.

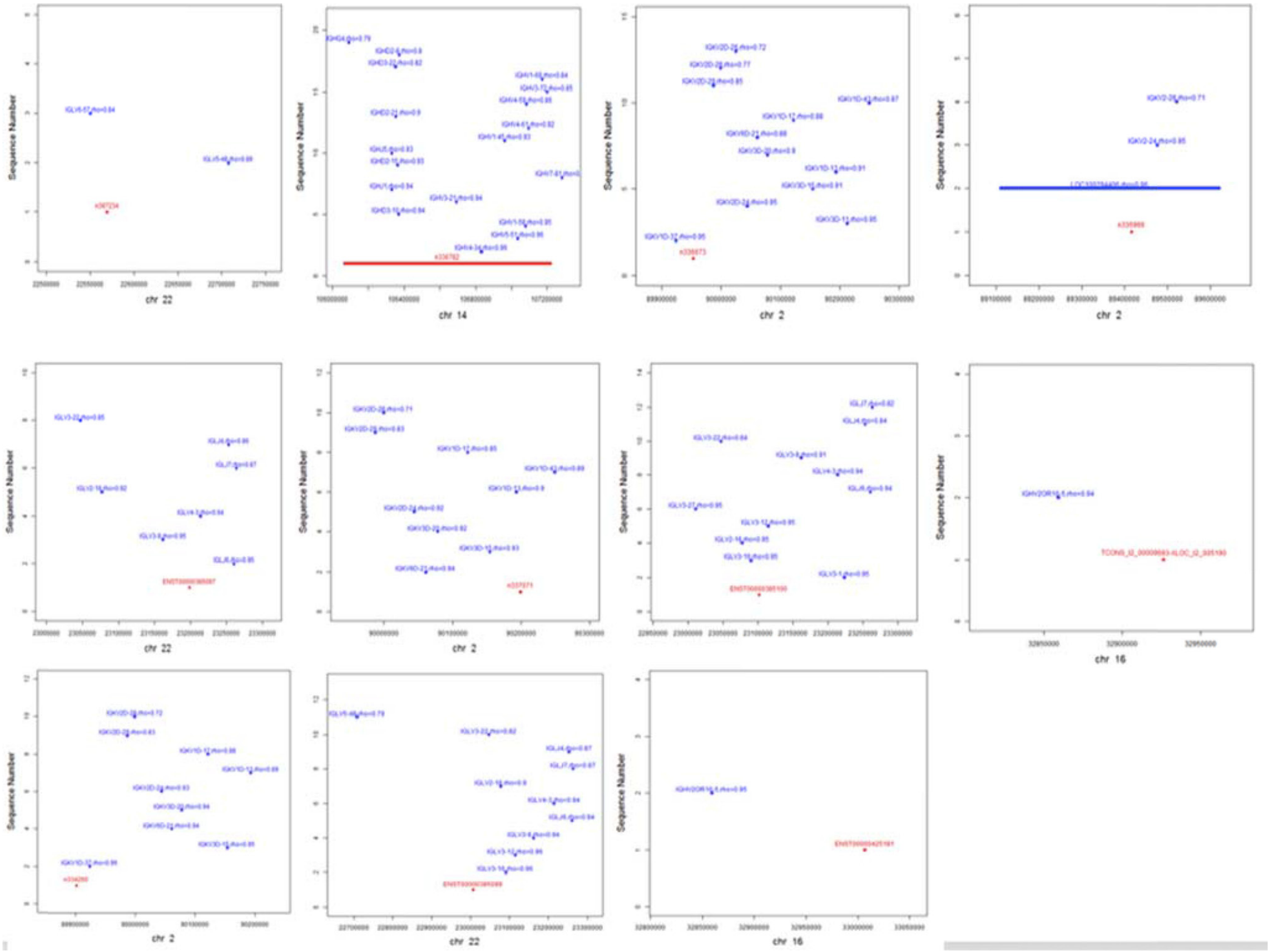

Supplementary Figure 4: Representative lncRNAs and the positions of their "cis-regulate" immunoglobulin genes in the chromosome.
